# Somaclonal Variation Is Induced *De Novo* via the Tissue Culture Process: A Study Quantifying Mutated Cells in *Saintpaulia*

**DOI:** 10.1371/journal.pone.0023541

**Published:** 2011-08-11

**Authors:** Mitsuru Sato, Munetaka Hosokawa, Motoaki Doi

**Affiliations:** Graduate School of Agriculture, Kyoto University, Sakyo-ku, Kyoto, Japan; United States Department of Agriculture, United States of America

## Abstract

**Background:**

The origin of somaclonal variation has not been questioned previously, i.e., “pre-existing mutations” in explants and “newly induced mutations” arising from the tissue culture process have not been distinguished. This is primarily because there has been no reliable molecular method for estimating or quantifying variation.

**Methodology/Principal Findings:**

We adopted a petal-variegated cultivar of *Saintpaulia* ‘Thamires’ (*Saintpaulia* sp.) as the model plant. Based on the difference between the pre- and post-transposon excision sequence of the promoter region of *flavonoid 3′, 5′-hydoroxylase* (*F3′5′H*), we estimated mutated (transposon-excised) cell percentages using a quantitative real-time PCR. Mutated cell percentages in leaf laminae used as explants was 4.6 and 2.4% in highly or low variegation flower plants, respectively, although the occurrences of blue color mutants in their regenerants were more than 40%. Preexisting mutated cell percentages in cultured explants were considerably lower than the mutated plant percentage among total regenerants via tissue culture.

**Conclusions/Significance:**

The estimation of mutated cell percentages became possible using the quantitative real-time PCR. The origins of mutations were successfully distinguished; it was confirmed that somaclonal variations are mainly caused by newly generated mutations arising from tissue culture process.

## Introduction

Tissue culture is a well known and efficient method of plant propagation, but the resulting regenerants often has a number of mutations termed “somaclonal variations” [1]. In the strict meaning of the term, somaclonal variations are thought to be derived from “newly induced mutations” arising from the tissue culture process as well as from “pre-existing mutations” in explants. Although some researchers have mentioned these two origins of somaclonal variations [2], they have been confused, and not enough attention has been paid to discriminate 1 from the other. However, as a premise for further research, the origins of somaclonal variations are important for ensuring the validity of this research. Generation of somaclonal variations is attributed to genetic and epigenetic modifications in DNA. In particular, transposable elements (TEs) are one of the causes of genetic rearrangements in *in vitro* culture. Tissue culture is reported to activate silent TEs, resulting in somaclonal variations [3].

**Figure 1:**
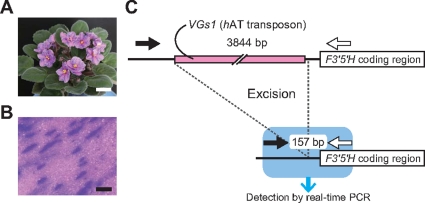
*Saintpaulia* ‘Thamires’ and the genetic background of a flower color change. (A) An original cultivar of ‘Thamires’. Bar  = 3 cm. (B) Magnification of a variegated petal. Bar  = 1,000 µm. (C) Schematic representation of the *F3′5′H* sequence in variegated ‘Thamires’ plants (not to scale). When the transposon is present (GenBank: AB596833), *F3′5′H* expression is disturbed, and cells become pink. On the other hand, when the transposon is excised (AB596834), *F3′5′H* expression resumes, and cells become blue. Arrows represent primers. In real-time PCR, only the post-excised sequence (157 bp) could be detected by shortening the elongation time.

The petal-variegated cultivar of *Saintpaulia* ‘Thamires’ (*Saintpaulia* sp.) shows flower color variants in the tissue culture-derived regenerants [4]. It was elucidated that the transposon *VGs1*: *h*AT superfamily plays pivotal roles in generating color variations, and blue cells in pink petals were generated by excising the TE (Fig. 1B) [4]. According to these results, PCR primers that could be used as genetic markers for detecting blue-colored cells were optimized for real-time PCR in order to quantify mutated cell percentages.

Real-time PCR is a conventional method used in biological research. It has been exploited to measure mRNA expression levels, DNA copy number, transgene copy number and expression, allelic discrimination, and viral titers [5]. As for the plant genome, real-time PCR has been frequently used to clarify the copy number of a particular gene in plant genomes [6]; however, the percentage of cells that have undergone sequence alterations had not been previously examined. In this study, we used a quantitative real-time PCR to measure sequence alterations caused by transposon excision.

In this study, we proposed an experimental system for quantifying the extent of mutations using a quantitative real-time PCR. This system enabled the estimation of mutated cell percentages in several plant parts, and the amount of newly induced mutations was determined by comparing the amount of pre-existing mutations in explants with the total amount of somaclonal variations. This system will provide the basis for the evaluation of mutated cell percentages in plants and enable further research into somaclonal variations.

## Results

### Results of leaf laminae cultures

Adventitious shoots were obtained from a medium variegated and a highly variegated plant via tissue culture (81 and 35 regenerants, respectively; Table 1). The most common mutation was solid blue mutants; in both cases, more than 40% plants were solid blue. Periclinal chimeras, which are composed of blue flower-color phenotype in L1 and variegated phenotype in L2 and L3, comprised around 10% of mutations and solid pink mutants comprised only a few percent. Among the regenerants, although variegated plants comprised ca. 40%, this value included miscellaneous plants with different extents of petal variegation, and the number of true-to-type plants (matching the parent phenotype) was less than 40%. As for the tissue culture results, no significant difference was observed between the two parents.

**Table 1:** Flower phenotypes of the leaf laminae culture-derived regenerants of *Saintpaulia* ‘Thamires’.

Parents	Flower phenotypes of regenerants				Total
	Variegation^a^	Solid blue	Periclinal chimera^b^	Solid pink	
Medium variegation^c^	44.4 (36)	44.4 (36)	9.9 (8)	1.2 (1)	100 (81)
Highly variegation	42.9 (15)	40.0 (14)	11.4 (4)	5.7 (2)	100 (35)

Parents differed in terms of the variegation intensity of petals. Numbers indicate proportion (%) of total regenerants, and numbers in parentheses indicate number of plants.

a Variegation intensity is not considered.

b These plants are thought to have a variegated L1 layer and blue L2 layer.

c This plant corresponds to the variegation intensity of the original cultivar.

### Validation of the real-time PCR system

**Figure 2:**
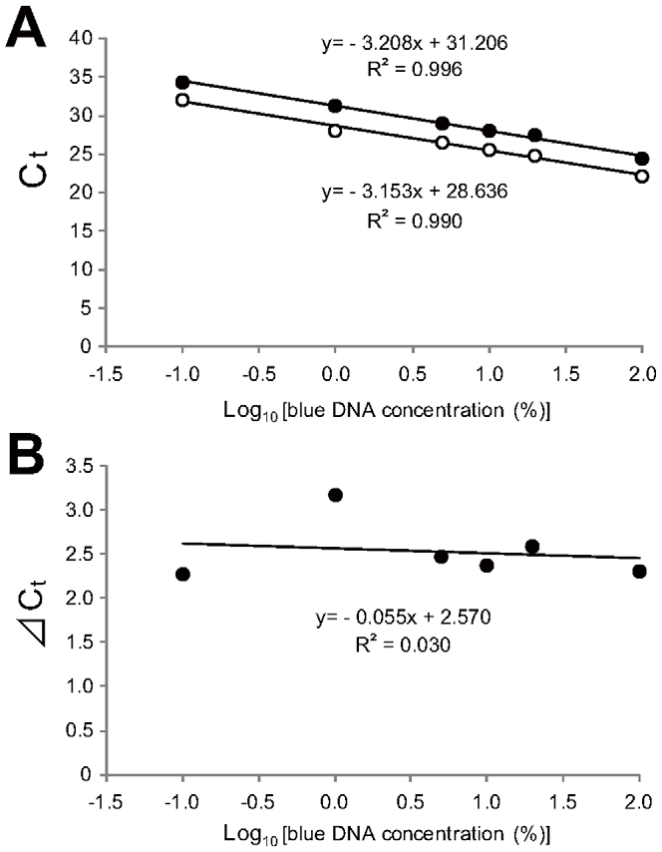
Comparison of amplification efficiencies and examination of the comparative C_t_ method within petal samples. (A) Standard curves obtained by a dilution series of petal DNA from solid blue mutants. Closed circles indicate the results for a post-transposon excision sequence (“target”), and open circles indicate the results for *CHS-A* (“reference”). (B) Difference between target C_t_ and reference C_t_ within petal samples. Slopes (0.055) smaller than 0.1 were valid (ABI User Bulletin #2). Identical procedures were used to validate the method in other plant parts (data not shown).

Based on the results of a previous report [4], real-time PCR primers were designed to detect post-transposon excision sequences only when the elongation time for PCR proess is shortend (Fig. 1). To determine the reference, amplification efficiencies of some primer sets for several candidate genes were investigated. The amplification efficiencies of each primer set were compared using a dilution series (data on petal samples are shown in Fig. 2; although the data for the other parts were omitted, similar results were obtained). Subsequently, a primer set for the *Chalcone synthase-A* (*CHS-A*) gene was adopted as reference. This process was required for applying comparative C_t_ methods.

**Figure 3:**
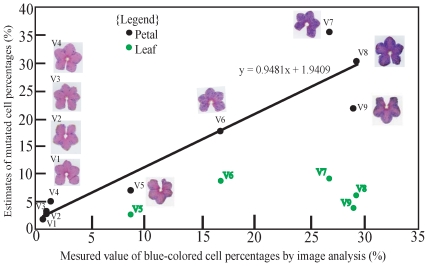
Comparison of estimates and measured values of mutated cell percentages. Data from 9 plants with different petal variegations are shown. For real-time PCR results, the average value for solid blue mutants was considered to be 100%, and values for other phenotypes are shown in comparison with those for solid blue mutants. Black circles represent the results for petals, and green circles represent the results for leaf laminae. Data for petals assessed using two different methods showed strong correlation (coefficient of correlation; 0.95), and these results validated the approach. In all cases, mutation rates in the leaf laminae were lower than those in the petals. Mutation rates in the leaf laminae of V1, V2, V3 and V4 were 0.34, 0.82, 0.46 and 0.94 respectively. Leaf laminae of V5 and V9 were used for *in vitro* shoot regeneration.

To assure the validity of this PCR system in the quantification of mutated cells, variegated petals were tested because the mutated cell percentages could be assessed by image analysis. The estimates of mutated cell percentages obtained using real-time PCR were compared with those obtained using the ImageJ software (NIH, USA; Fig. 3). Solid blue petals were selected to comprise 100% mutated cells (used as a calibrator), and no amplification of PCR was detected from solid pink petals. The slope of the regression line between the data of image analysis and real-time PCR for 9 different plants was 0.9481 (Fig. 3). This value is close to 1.0 of the theoretical value. Data from these two methods showed strong correlation (coefficient of correlation was 0.95) and confirmed the validity of the approach.

### Mutated cell percentages in leaf laminae used for *in vitro* culture

Mutated cell percentages of medium variegated petals were estimated to be 8.6% by image analysis and 6.7% by real-time PCR; the mutated cell percentages of highly variegated petals were estimated to be 28.9% by image analysis and 22.0% by real-time PCR. Mutated cell percentages of the leaf laminae used as explants in tissue culture were assessed: 2.2% mutation was observed in a medium variegated plant, 4.6% in an highly variegated plant, and 8.7% in the variegated plant (Fig. 3). The mutated cell percentages of leaf laminae estimated by real-time PCR were always lower than those of petals. Mutated cell percentages in leaves of the strains with highly variegation (V7, V8 and V9) were a little higher than those of the strains with low variegations.

**Figure 4:**
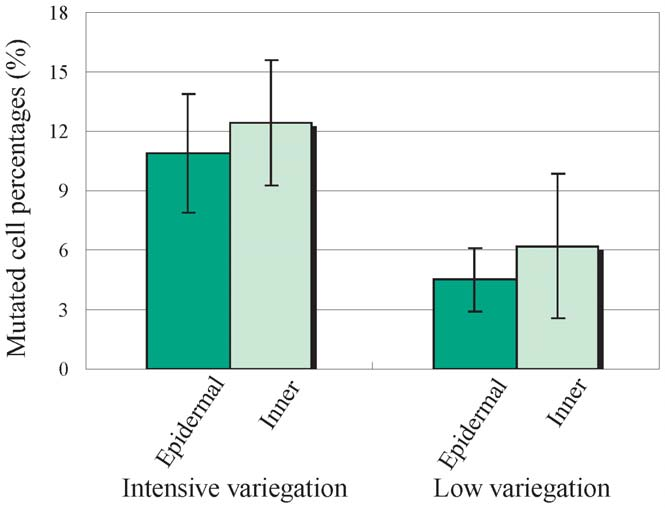
Mutated cell percentages in different layers of the petiole in the two variegated types. Mutated cell percentages were calculated by real-time PCR analysis. The average value for solid blue mutants (whole petiole was examined) was considered to be 100%, and values for other phenotypes are shown in comparison with those for solid blue mutants. V6, V7 and V8 plants were used for highly variegated plants and V1, V2, V3 and V4 plants were used for low variegated plants. Data presented as mean ± standard deviation. No statistical differences were not observed between epidermal and inner layers in both variegated types.

To test the hypothesis of the localization of mutated cells, the mutated cell percentages of different cell layers were assessed. The extracted DNAs from tissues with epidermis (L1) and without epidermis (L2+L3) were tested separately. Significant differences were not observed by *t*-test at 0.05% level between these two tissues (Fig. 4).

### Mutated cell percentages in other plant parts

**Figure 5:**
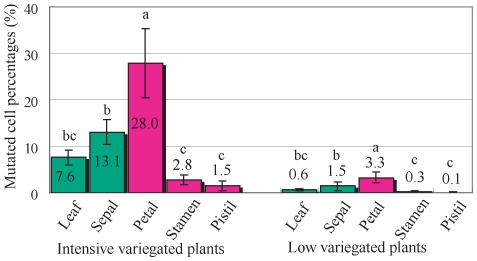
Mutated cell percentages in different parts of the two variegated types. Variegated areas of petals were determined by image analysis in highly and low variegated plants. Real-time PCR analysis was used to determine mutated cell percentages. The average value for solid blue mutants (whole petiole was examined) was considered to be 100%, and values for other phenotypes are shown in comparison with those to solid blue mutants are shown. V6, V7 and V8 plants were used for highly variegated plants, and V1, V2, V3 and V4 plants were used for low variegated plants. Data presented as mean ± standard deviation. Different letters indicate statistical significance (*P*<0.05) as determined by ANOVA followed by Ryan's test.

In addition, using real-time PCR analysis, the mutated cell percentage in other plant parts of two different variegation types was investigated. Among the parts tested here, petals showed the highest mutation rates (Fig. 5), followed by sepals and leaves. Stamens and pistils showed relatively low mutation rates (Fig. 5). The mutated cell percentages of each organ of highly variegated plants were higher than those of the low variegated plants.

## Discussion

### Solid blue mutants as the dominant mutants of somaclonal variations of leaf laminae culture

Leaf laminae from two different plants with different variegation intensities in their petals were cultured (Table 1). In the tissue cultures mentioned above, approximately 40% progenies were solid blue mutants, and there was no significant difference between the progenies from the two parents (Table 1). Mutation rates including periclinal chimera and solid pink plants were more than 50%, and in addition these mutants, other minor variations such as altered-variegation mutants should be considered for total mutation rates. However, the most frequently observed mutants were solid blue, and this study aimed to determine the tendency toward somaclonal variations by focusing solid blue mutants alone. The fact that the variegation intensity of the parents did not affect mutation rates implied that pre-existing mutations in explants did not have a significant impact on the occurrence of somaclonal variations.

### Establishment of a method for quantifying mutated cell percentages

To evaluate the amount of mutated cells using numerical data, solid blue mutants were utilized as mutation indicators. In this experiment, primer sets for a quantitative real-time PCR system were optimized, and the adjustment of the elongation time enabled detection of a single post-excision sequence (Fig. 1). As shown in Fig. 3, a validation experiment using variegated petals was successful; the mutated cell percentage assessed by image analyses was strongly correlated with the estimates of mutated cell percentages obtained by real-time PCR. However, image analysis can not detect very small splotches on the petals, which will lead a devious result as V7 on Fig. 3. And when the splotches localized in a particular area in a petal as V9, image analysis was liable to overestimate the blue-colored cell percentages. Although such errors may not seriously affect the results of this study, the method used for image analysis could be further improved by arranging the parameters of ImageJ and by fixing on the stages suitable for this software.

### The distribution of mutated cells in leaf laminae and the origin of somaclonal variation

**Figure 6:**
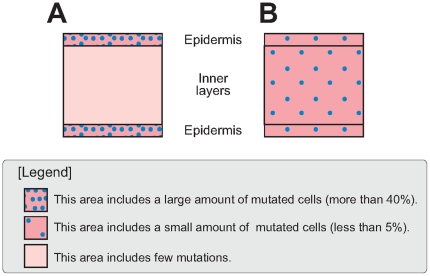
Illustration of 2 conflicting models of the distribution of mutated cells. This figure represents the longitudinal section of a leaf lamina, and the intensity of dots represents the frequency of mutation, or transposon excision. In model A, the epidermis contains a large amount of mutated cells, and the inner layers contain few mutations. In model B, the mutation rate is not high, but mutated cells are homogenously distributed. We observed that model B was more suitable for explaining the results shown in Figure 4.

Using the aforementioned method, mutated cell percentages in leaf laminae were assessed (Fig. 3). The mutated cell percentage was less than 10% in every case and was much lower than the mutation rates observed in the tissue culture results (Table 1). However, it was not possible to confirm whether the “real” mutation rates in leaf laminae were low since high mutation rates exhibited by the tissue culture results could be explained by the localization of mutated cells; if transposon-excised cells were localized in epidermal cell layers, mutation rates in the leaf laminae would appear to be low (Fig. 6A). In case of *Saintpaulia*, shoots are always generated from the epidermis [7]–[9]. Considering that shoots were regenerated only from the epidermis in tissue culture, the high mutation rates indicated from the tissue culture results supported this hypothesis. However, to investigate this, DNA isolation separately from the epidermal and inner cell layers was essential. Because it was extremely difficult to peel the epidermis from the leaf lamina, petioles were used, and the dissected epidermal and inner cell layers were used for DNA isolation and real-time PCR analysis. The analyses were conducted using petioles from medium and highly variegated plants, no significant between-group difference was detected (Fig. 4). Therefore, localization of mutated cells was deemed unlikely, refuting model A and supporting model B (Fig. 6).

**Figure 7:**
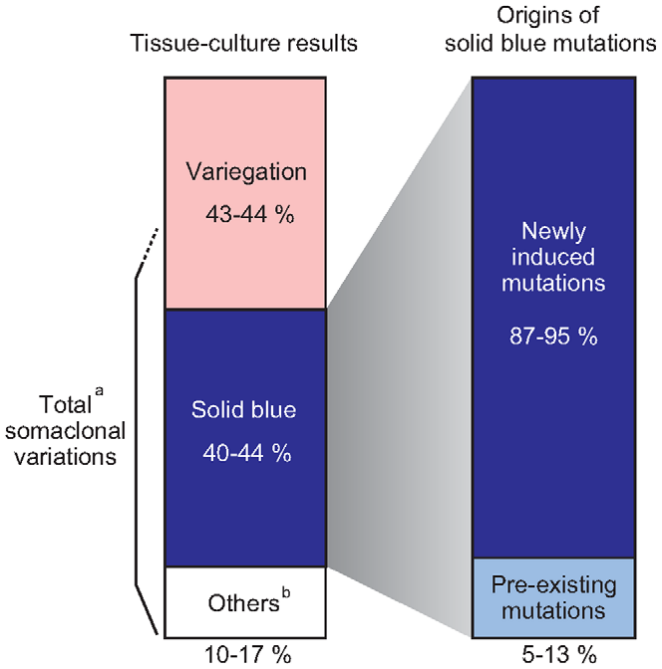
Tissue culture results (left) and origins of solid blue mutations (right). Percentages were calculated based on the results of medium and highly variegated plants. Solid blue was the most frequent form of somaclonal variations of ‘Thamires.’ Most of these variations were derived from newly induced mutations arising from the tissue culture process. The numbers in this figures are calculated by the result of Table 1.^a^Total number of somaclonal variations should be include solid blue mutants, other phenotypes, and altered-variegation mutants. ^b^Others include periclinal chimeras and solid pink mutants.

In summary, it is suggested that most somaclonal variations were derived from newly generated mutations arising from the tissue culture process and not pre-existing mutations in the explants (Fig. 7). Mutated cell percentages remained low in the explants but increased rapidly during the tissue culture.

### Detection or monitoring of somaclonal variations and application of the method in further studies

In recent years, molecular biological approaches have been utilized for detecting somaclonal mutations [3], [10], [11], including amplified fragment length polymorphisms [12]–[18] and inter-simple sequence repeats [13], [19]–[22]. These methods are preferable for detecting unidentified or unspecified mutations and determining whether a mutation has occurred. However, once the background of the mutation is specified (transposon excision in this case), further research requires other analytical tools such as quantification of mutations. Real-time PCR requires only a small amount of DNA, and has high reproducibility. The method proposed here will be applicable to variety of research studies.

Using this method, we analyzed the distribution of mutated cells in each plant component. The parts that suffer from transposon excision most frequently are the petals (Fig. 5). The mutated cell percentage observed in the leaf laminae and sepals was lower than the mutation rate observed in petals. In addition, anthers and styles showed relatively low mutation rates (Fig. 5). The reason for this difference remains unclear and will need to be determined in future. The hypothesis that a certain mechanism suppresses transposon excision may be accurate. In the context of sexual reproduction, such a mechanism may work in reproductive organs to ensure reasonable genetic stability, but no such data have been obtained until date.

The triggers of mutations in tissue culture remain controversial [23]; numerous factors have been suggested, including plant growth regulators, lighting conditions [24], aseptic conditions, imbalances of media components, the relationship between high humidity and transpiration [25], [26], saline stress, oxidative stress and nutrient deficiency [23]. It is clear that some factors stimulate transposon movement in the tissue culture process. Because a method for quantifying mutations in *Saintpaulia* ‘Thamires’ has been established, it may be possible to elucidate the specific causes of somaclonal variations.

## Materials and Methods

### Plant materials

*Saintpaulia* ‘Thamires’ (*Saintpaulia* sp.) was obtained from Royal Green Inc. (Gifu, Japan). These plants were grown in a greenhouse at the Experimental Farm of Kyoto University (Kyoto, Japan). The greenhouse was shaded to allow a light intensity of 140–200 µmol·m^−2^·s^−1^ and was maintained at an appropriate temperature below 35°C with mist cooling in summer and above 10°C with oil heating in winter.

Nine different plants with different variegation levels were selected from *in vitro* cultured regenerants for experiments. And two different plants showing different extents of petal variegation were used for *in vitro* shoot regeneration (V5 and V9, medium and highly variegated plants, respectively). Leaf laminae were harvested from plants and sterilized with 0.5% sodium hypochlorite for 7 min; leaf discs (8 mm diameter) were then excised as explants and placed on modified Murashige and Skoog (MS) medium [27] supplemented with 1 ppm each of α-naphthaleneacetic acid and 6–benzylaminopurine, 3% (w/v) sucrose, and 0.3% (w/v) gellan gum (Wako, Japan). The medium pH was adjusted to 5.8–6.0 prior to autoclaving at 121°C for 15 min. A few months later, the adventitious shoots generated were excised and transferred to a half-strength MS medium without plant hormones, supplemented with 1% (w/v) sucrose, and 0.3% (w/v) gellan gum. All plantlets were grown in a growth chamber maintained at 25°C under 16-h photoperiod conditions and a light intensity of 40 µmol m^−2^ s^−1^ provided by cool white fluorescent lights. These plantlets were then transferred to vermiculite and acclimatized under intermittent misting conditions. Next, they were transferred to peat moss based soil and grown under greenhouse conditions. When the plants flowered, their phenotypes were assessed.

### Isolation of nucleic acids

From the tissue culture derived progenies, several plants with different extents of variegation on their petals were selected and used for DNA isolation. DNA was isolated from fully-opened petals, leaf laminae, petioles, stalks, sepals, anthers, and styles of variegated plants and solid pink and blue mutants using a DNeasy® plant mini kit (QIAGEN, Germany) in accordance with the manufacturer's instructions. To assess the localization of mutated cells based on cell layers, petioles were dissected vertically into the outer and inner layers, that is, with and without epidermis and DNA was isolated separately.

### Real-time PCR

Based on sequence differences [4], primers were designed for detecting a post-transposon excision sequence (“target”) on the promoter region of *flavonoid 3′, 5′-hydoroxylase* (*F3′5′H*). As a “reference,” several primer sets for the candidate genes [*Actin* (GenBank accession number: AB596843), *CHS-A* (DQ788862) and *STM2* (AY662094) from reported data, *bHLH* and *F3′H* from our unpublished data] were tested to determine their amplification efficiencies. With a dilution series of DNA from solid blue mutants, standard curves were obtained by real-time PCR. By comparing amplification efficiencies, an optimized combination of primer sets was selected to validate the ΔΔC_t_ (comparative C_t_) method; the primers used to detect the post-transposon excision sequence were Up-73 Fw (5′-ACACATGCAATGCTGCTCTATTA-3′) and Code-84 Rev (5′-ACAGAAATTTCCGGATGATGAAG-3′), those used todetect the *CHS-A* reference gene were CHS-A Fw (5′-CTCTTGAAGGATGTCCCTTCTTT-3′) and CHS-A Rev (5′-TTCAACTCAACCTGATCCAAGAT-3′). Real-time PCR was performed using a 7900HT thermal cycler (Applied Biosystems, USA) with the SDS 2.1 software (Applied Biosystems, USA). The PCR mixture contained 2 µl of template gDNA (corresponding to 1–2 ng), 10 µl of SYBR Premix Ex Taq II (TaKaRa, Japan), and 0.2 µM of forward and reverse primers in a final volume of 20 µl. The program was set at 95°C for 10 s, followed by 40 cycles at 95°C for 5 s and at 60°C for 10 s; melting curves were inspected visually in the subsequent dissociation steps (95°C for 15 s, 60°C for 15 s, and 95°C for 15 s).

### Data analyses by the ΔΔC_t_ (comparative C_t_) method

For the quantification of mutated cell percentages, the number of PCR cycles (C_t_) to reach the threshold level was analyzed with the ΔΔC_t_ (comparative C_t_) method (according to ABI User Bulletin #2) [28], [29]. ΔC_t_ is the difference in the threshold cycle of the target and reference genes. As calibrator samples, genomic DNA from solid blue mutants were used because these were presumed to consist of 100% mutated cells. ΔΔC_t_ was calculated by ΔC_t_ minus the calibrator C_t_, and ΔΔC_t_ values were compared with those from the equivalent samples of solid blue mutants (i.e., the ΔΔC_t_ of the variegated petal was compared with the ΔΔC_t_ of the blue petals). More than three replicates of each sample were used, and average values were obtained.

### Image analysis

Fully opened petals were sampled from variegated plants and dissected petals were scanned using the digital scanner GT-9800F (EPSON, Japan). Scanned images were processed using the image analysis software ImageJ (NIH, USA). Color images were first split into RGB images. The variegated or blue-colored cell area was specified in a blue channel image by manually adjusting threshold levels and area was measured by “analyzing particles”. In addition, the total area of the petals was measured in a red channel image, and the percentage of blue cells was calculated by comparing the area of blue cells to the total area of the petals.

### Statistical analysis

ANOVA was performed using the ANOVA4 software, and Ryan's test was subsequently performed as a *post-hoc* test. Student's *t*-test was applied to the data to evaluate differences in mutated cell percentages of epidermal and inner tissues of petioles. The Excel 2007 software was used for correlative analysis and Student's *t*-test. A significance level of *P*<0.05 was considered for all analyses.
